# Team-based care for improving hypertension management among outpatients (TBC-HTA): study protocol for a pragmatic randomized controlled trial

**DOI:** 10.1186/s12872-017-0472-y

**Published:** 2017-01-21

**Authors:** Valérie Santschi, Grégoire Wuerzner, Arnaud Chiolero, Bernard Burnand, Philippe Schaller, Lyne Cloutier, Gilles Paradis, Michel Burnier

**Affiliations:** 1 La Source School of Nursing Sciences, University of Applied Sciences Western Switzerland, Av. Vinet 30, 1004 Lausanne, Switzerland; 2 Service of Nephrology and Hypertension, Lausanne University Hospital, Lausanne, Switzerland; 3 Institute of Social and Preventive Medicine, Lausanne University Hospital, Lausanne, Switzerland; 4 Cité Générations, Onex, Switzerland; 5 Département des Sciences Infirmières, Université du Québec à Trois-Rivières, Trois-Rivières, Canada; 6 Department of Epidemiology, Biostatistics, and Occupational Health, McGill University, Montreal, Canada

**Keywords:** Hypertension, Team-based care, Collaboration, Healthcare professionals, Healthcare services, Intervention

## Abstract

**Background:**

Blood pressure (BP) is poorly controlled among a large proportion of hypertensive outpatients. Innovative models of care are therefore needed to improve BP control. The Team-Based Care for improving Hypertension management (TBC-HTA) study aims to evaluate the effect of a team-based care (TBC) interprofessional intervention, involving nurses, community pharmacists and physicians, on BP control of hypertensive outpatients compared to usual care in routine clinical practice.

**Methods/design:**

The TBC-HTA study is a pragmatic randomized controlled study with a 6-month follow-up which tests a TBC interprofessionnal intervention conducted among uncontrolled treated hypertensive outpatients in two ambulatory clinics and among seven nearby community pharmacies in Lausanne and Geneva, Switzerland. A total of 110 patients are being recruited and randomized to TBC (TBC: *N* = 55) or usual care group (UC: *N* = 55). Patients allocated to the TBC group receive the TBC intervention conducted by an interprofessional team, involving an ambulatory clinic nurse, a community pharmacist and a physician. A nurse and a community pharmacist meet patients every 6 weeks to measure BP, to assess lifestyle, to estimate medication adherence, and to provide education to the patient about disease, treatment and lifestyle. After each visit, the nurse and pharmacist write a summary report with recommendations related to medication adherence, lifestyle, and changes in therapy. The physician then adjusts antihypertensive therapy accordingly. Patients in the UC group receive usual routine care without sessions with a nurse and a pharmacist. The primary outcome is the difference in daytime ambulatory BP between TBC and UC patients at 6-month of follow-up. Secondary outcomes include patients’ and healthcare professionals’ satisfaction with the TBC intervention and BP control at 12 months (6 months after the end of the intervention).

**Discussion:**

This ongoing study aims to evaluate the effect of a newly developed team-based care intervention engaging different healthcare professionals on BP control in a primary care setting in Switzerland. The results will inform policymakers on implementable strategies for routine clinical practice.

**Trial registration:**

ClinicalTrials.gov registration: NCT02511093. Retrospectively registered on 28 July 2015.

## Background

High blood pressure (BP) is a major risk factor for cardiovascular diseases (CVD) and mortality worldwide [1]. Although treatment of hypertension can substantially reduce this risk, hypertension remains underdected, undertreated, and poorly controlled [2, 3]. For example, half of North American treated patients with hypertension remain uncontrolled [4, 5]. A similar proportion has been found in Switzerland [6]. Furthermore, due to ageing populations, busy clinical workloads, and shortage of physicians in most healthcare systems, new approaches to hypertension care, involving pharmacists [7, 8] or nurses [9, 10], could be a promising approach to improve BP management and control.

Pharmacists are highly accessible healthcare professionals and indeed a valuable asset to improve hypertension management by providing medication management in collaboration with physicians and by supporting patients in medication intake [11–14]. Evidence supports that pharmacists – working alone or in teams – are effective for the management of hypertension [13, 15–17] and other CVD risk factors [16, 18, 19]. Nurses, by providing lifestyle counseling and health education, are also helpful for the management chronic diseases [20, 21], including hypertension [9, 22–24]. They are a valuable member of team-based care at the interface between patients and physicians [25]. Furthermore, nurses can also intervene in collaboration with pharmacists to improve BP as shown in a Canadian study and in community-based prevention programs in Canada and in the USA [26–30]. Santschi et al. demonstrated that a collaborative model involving community pharmacists and primary care physicians focused on the management of drug adherence was feasible in the Swiss healthcare system [31] and improved long-term BP control among uncontrolled hypertensive patients [32].

Team-based care is a coordinated model of care involving different healthcare professionals, such as physicians, pharmacists, nurses or other non-physician clinicians, working in a collaborative partnership, each with their own expertise [18, 25, 27]. Team-based care of hypertension has recently been recommended by the U.S Community Preventive Services Task Force [33, 34]. To this day, a team-based care, involving nurses and community pharmacists working in collaboration with physicians, to improve BP control need to be evaluated in European countries, and in particular in Switzerland.

Therefore, we launched the Team-Based Care for improving Hypertension management (TBC-HTA) randomized controlled study. This study is designed to evaluate if a TBC interprofessional intervention, involving nurses and community pharmacists working in collaboration with physicians, improves BP control among uncontrolled treated hypertensive patients under real-life conditions.

## Methods/design

### Study design and setting

**Fig. 1:**
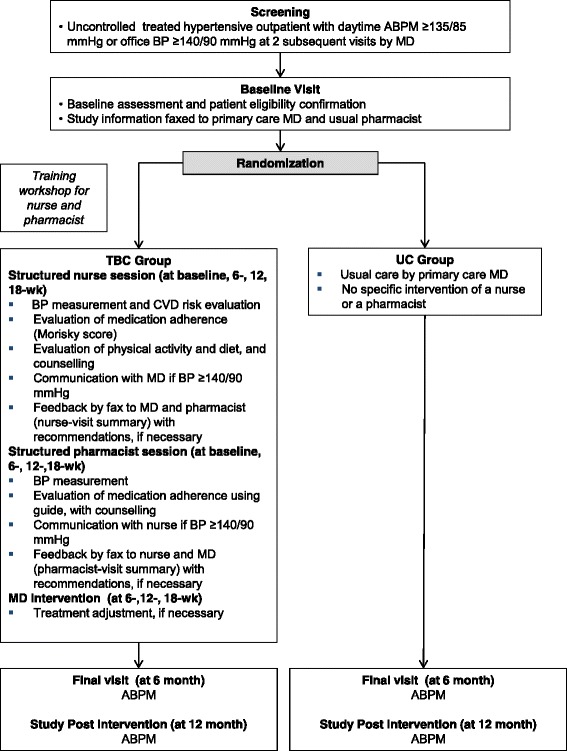
Study Flow Diagram

The TBC-HTA study is an ongoing 3-year multicenter pragmatic randomized controlled trial comparing a 6-month team-based care interprofessional intervention, involving nurses, community pharmacists and physicians, to a usual care group among 110 outpatients followed in ambulatory clinics and their nearby community pharmacies in Lausanne and Geneva areas, Switzerland (Fig. 1). The patient is the unit of randomization and the unit of analysis. We applied a pragmatic approach to determine the effect of the TBC intervention under real-life conditions with existing community healthcare professional resources [35].

Treated uncontrolled hypertensive outpatients followed in ambulatory clinics are recruited and randomly allocated to one of two groups: 1) the TBC intervention group (TBC: *N* = 55), in which patients receive care from nurses and community pharmacists working in collaboration with physicians; 2) the usual care group (UC: *N* = 55) in which patients receive routine care without any intervention from nurses or community pharmacists. Patients are recruited from two ambulatory clinics: 1) the Hypertension Clinic, an outpatient clinic affiliated with Lausanne University Hospital (CHUV; www.chuv.ch) and located in Lausanne, 2) Cité générations, an ambulatory care center located in Geneva (www.cite-generations.ch). Regular staff nurses and physicians of the Hypertension Clinic and Cité Générations are involved in the study. Nearby community pharmacists in Lausanne and Geneva are recruited based on their geographical proximity to the ambulatory clinics to facilitate the follow-up of patients.

### Participants

#### Identification and recruitment

Patients with the following inclusion criteria are eligible to participate: 1) uncontrolled hypertension [defined as daytime systolic ambulatory blood pressure measurement (ABPM) ≥135 and/or diastolic ABPM ≥85 mmHg or office systolic BP ≥140 and/or office diastolic ≥90 mmHg over at least two consecutive visits [36]]; 2) taking at least one antihypertensive medication; 3) aged 18 years old or more; 4) speak and understand French; and 5) agree to use the service from the same pharmacy for the duration of the study. Patients are excluded if they 1) are unable to understand the study aim; 2) are pregnant or lactating; 3) live in a nursing home; 4) are hospitalized; 5) participate in another study; or 6) have daytime 24-h ABPM > 180/110 mmHg. Eligible patients are contacted by phone by a nurse who explains the study and ascertains the patient’s willingness to participate. If the patient agrees to participate, the study information material is sent and an appointment is scheduled by the nurse at the ambulatory clinics. After consenting and completing the baseline assessment, patients are randomized in a 1:1 allocation ratio to the TBC intervention group or to the UC care group.

### TBC intervention

The TBC interprofessional intervention, based on distinct competencies of healthcare professionals involved in hypertension care, comprises:

1. A 2-h training workshop during which nurses and community pharmacists are trained about the study requirements, standardized BP measurement and hypertension care according to the European Society of Hypertension recommendations [36], antihypertensive medication management (including the assessment of medication adherence), and counseling about lifestyle modification (physical activity and diet);
2. Structured individual sessions conducted by ambulatory clinic nurses at baseline, 6, 12 and 18-week and structured individual sessions conducted by community pharmacists at baseline, 6, 12 and 18-week. Specifically, at each session, the nurse measures BP, estimates adherence using the Morisky Medication Adherence Scale (MMAS-8) [37, 38], and provides lifestyle counseling (physical activity and diet) during structured face-to-face interviews with the patient. After each session, the nurse sends a summary report (outlining BP measurements, score of MMAS-8, physical activity and diet assessment with any counselling and recommendations) is sent by fax to the pharmacist. The physician has access to this report. The patient is then referred to the community pharmacist who measures BP and emphasizes medication adherence with the patient (using a specified guide following a step process: gathering information from the patient, creating a medication list, and identifying drug related problems) during each structured individual session. After each session, the pharmacist sends a summary report (outlining BP measurement, score of MMAS-8, and any recommendations to change treatment) by fax to the nurse. The physician has access to this report.

No medication change is allowed during the first 6 weeks of follow-up. If BP is uncontrolled (≥140/90 mmHg) at the 6, 12 and 18-week session with the community pharmacist or the nurse, a contact (by phone or face-to-face) with the physician is made by the nurse. Taking account of the nurses’ and community pharmacists’ recommendations on lifestyle, medication adherence, and therapy, the physician adapts the treatment if necessary.

### Usual care group

Patients in the UC group received routine care by their usual physician without nurse or community pharmacist intervention.

### Blood pressure measurement

At each visit, BP is measured in TBC patients by the nurse and the community pharmacist using the clinically validated Microlife WatchBP home oscillometric device [39], using a standardized protocol. At the end of the 6-month follow-up, ABPM is performed among TBC and UC patients using the clinically validated electronic Diasys device (DIASYS integra; Novacor SA, Rueil-Malmaison, France) [40]. The ABPM device is installed on the dominant arm by the nurse who explains the procedure to the patient. Measurements are based on the auscultatory mode, relayed by the oscillometric mode in case of failure of the auscultatory mode. Measurements are made every 20-min intervals during the day and every 60-min intervals during the night [36]. The mean daytime ABPM is calculated from the average of BP readings obtained between 9.30 am to 9.30 pm.

### Outcomes

The primary outcomes are 1) the difference in mean daytime ABPM at 6-month between TBC and UC patients and 2) the difference in the proportion of patients with controlled BP (daytime ABPM <135/85 mmHg) at 6-month between TBC and UC patients. Secondary outcomes include 1) patient’s and healthcare professionals’ satisfaction with the TBC intervention (using specified questionnaires administered at the end of the follow-up); 2) the difference in the proportion of TBC and UC patients with controlled BP (daytime ABPM <135/85 mmHg) at 12 months (6 months after the end of the intervention).

### Sample size

Based on the results of our systematic review assessing the impact of pharmacist interventions on BP [41], a difference in systolic BP of 6 to 10 mmHg is expected between TBC and UC care groups at the end of 6 months of follow-up. A total of 46 patients per group provides 80% power to detect a 6 mmHg difference in systolic BP (SD: 10 mmHg) at 6-month of follow-up with a two-sided alpha of 5%. Assuming a drop-out or loss to follow-up rate of ~15%, the sample size is adjusted to 55 per group, for a total sample size of 110.

### Randomization and blinding

Participants are randomized via a computer number generator using sequentially numbered opaque sealed envelopes in a 1:1 allocation ratio to either TBC or UC group, using permuted blocks. The block sizes will not be disclosed, to ensure concealment of allocation. Assignments were made in advance by a statistician who has prepared the sequentially numbered opaque sealed envelopes that contain the randomization assignment. Separate lists of randomization are produced for each clinic. Numbered envelopes are opened after obtaining each patient’s consent.

Study investigators, co-investigators and collaborators are not informed of the randomization sequence. Due to the nature of the intervention, patients and healthcare professionals (physicians, nurses and community pharmacists) cannot be blinded to the intervention.

### Statistical analyses

All analyses will be conducted following the intention-to-treat principle. The statistician will be blinded to group allocation. Baseline characteristics of the TBC and UC groups will be assessed via descriptive analyses in terms of age, gender, co-morbidities, smoking status, body mass index, BP and prescribed medications. Baseline characteristics of nurses, pharmacists and physicians will be also described, such as age, 6, year of graduation, and pharmacist status (pharmacy owner, salaried employee). Means (standard deviations) and proportions will be computed for continuous and categorical variables, respectively.

Mean systolic and diastolic BP at month-6 will be compared between the study groups using a Student’s two-sided *t*-test. At 6-month, the proportion of patients with BP control (daytime ABPM <135/85 mmHg) will be compared between the two groups using a chi-squared test. To account for potential imbalances between groups, multivariate linear or logistic regression will be used to compare mean BP or odds of controlled BP at 6 months between the TBC and UC groups, respectively. Two-sided P value less than 0.05 will be considered as statistically significant. Analyses will be made with Stata 13.0 software (StataCorp LP).

## Discussion

The TBC-HTA randomized controlled study is ongoing and will be the first attempt to evaluate a new approach focused on a team-based care intervention engaging different healthcare professionals to improve BP control in a primary care setting in Switzerland. The results of the current study will complete knowledge in the team-based care approach but also inform policymakers on implementable strategies in the future. Indeed, this study could make a significant contribution to various healthcare systems in terms of efficacy and possible implementation and dissemination of a collaborative approach for hypertension management.

There are potential limitations to this study. Only 2 ambulatory clinics participate in the study. In addition, these ambulatory clinics were selected because they were interested in implementing the TBC interprofessional intervention in their clinical practice. This could limit the external validity of the study. However, the fact that the study is conducted under real-life conditions with existing community healthcare professional resources, increases the generalizability and the possibility of disseminating the intervention (if effective) in primary care settings.

The value of this study could be enhanced by an economic analysis, integrating an estimate of patient contact time by the nurses, community pharmacists and physicians. This analysis could offer policy makers compelling arguments for the dissemination of the TBC intervention. Therefore, as secondary and exploratory analyses, we will make some estimations of the cost of the intervention.

Although the Swiss healthcare system has characteristics which may hidden the generalizability of our findings, our results combined with those of other similar studies should provide impetus for the gradual dissemination of the TBC model to other settings and countries with a high burden of hypertension and shortage of physicians.

Strengths of the TBC-HTA study include its design as a RCT and the use of existing community healthcare professional resources in real-life conditions to evaluate the collaborative approach. In addition, TBC model of hypertension care could offer opportunities for efficient approach to hypertension treatment and control in real-life conditions and will inform policy makers on strategies to implement.

## Abbreviations

RCT: Randomized controlled trial
TBC: Team-based care
